# Fabrication of Flexible and Transparent Metal Mesh Electrodes Using Surface Energy‐Directed Assembly Process for Touch Screen Panels and Heaters

**DOI:** 10.1002/advs.202304990

**Published:** 2023-10-11

**Authors:** Siqing Yuan, Zebin Fan, Guangji Wang, Zhimin Chai, Tongqing Wang, Dewen Zhao, Ahmed A. Busnaina, Xinchun Lu

**Affiliations:** ^1^ State Key Laboratory of Tribology in Advanced Equipment Tsinghua University Beijing 100084 China; ^2^ Department of Mechanical Engineering Tsinghua University Beijing 100084 China; ^3^ NSF Nanoscale Science and Engineering Center for High‐Rate Nanomanufacturing (CHN) Northeastern University Boston Massachusetts 02115 USA

**Keywords:** directed assembly, flexible and transparent electrodes, silver nanoparticles, surface energy, wetting/dewetting

## Abstract

Transparent conductive electrodes (TCEs) are indispensable components of various optoelectronic devices such as displays, touch screen panels, solar cells, and smart windows. To date, the fabrication processes for metal mesh‐based TCEs are either costly or having limited resolution and throughput. Here, a two‐step surface energy‐directed assembly (SEDA) process to efficiently fabricate high resolution silver meshes is introduced. The two‐step SEDA process turns from assembly on a functionalized substrate with hydrophilic mesh patterns into assembly on a functionalized substrate with stripe patterns. During the SEDA process, a three‐phase contact line pins on the hydrophilic pattern regions while recedes on the hydrophobic non‐pattern regions, ensuring that the assembly process can be achieved with excellent selectivity. The necessity of using the two‐step SEDA process rather than a one‐step SEDA process is demonstrated by both experimental results and theoretical analysis. Utilizing the two‐step SEDA process, silver meshes with a line width down to 2 µm are assembled on both rigid and flexible substrates. The thickness of the silver meshes can be tuned by varying the withdraw speed and the assembly times. The assembled silver meshes exhibit excellent optoelectronic properties (sheet resistance of 1.79 Ω/□, optical transmittance of ≈92%, and a FoM value of 2465) as well as excellent mechanical stability. The applications of the assembled silver meshes in touch screen panels and thermal heaters are demonstrated, implying the potential of using the two‐step SEDA process for the fabrication of TCEs for optoelectronic applications.

## Graphical abstract

A two‐step surface energy‐directed assembly (SEDA) process is developed to efficiently fabricate high resolution (down to 2 µm) silver meshes on both rigid and flexible substrates. The thickness of the silver meshes can be tuned by varying the withdraw speed and the assembly times. The applications of the silver meshes in touch screen panels and thermal heaters are demonstrated.

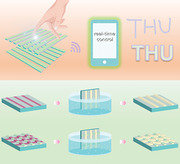

## 1 Introduction

With the advent of the information age, optoelectronic devices including displays (liquid crystal displays (LCDs)^[^ ^1^ ^]^ and light‐emitting diodes (LEDs)^[^ ^2^, ^3^ ^]^), touch screen panels,^[^ ^4^, ^5^, ^6^, ^7^, ^8^ ^]^ solar cells,^[^ ^9^, ^10^, ^11^ ^]^ smart windows,^[^ ^12^ ^]^ etc. are increasingly used in our daily lives, continuously enriching and facilitating our lives. Transparent conductive electrodes (TCEs) are indispensable components of these optoelectronic devices, transmitting light and transporting charges. To date, transparent and conductive metal oxides, particularly indium tin oxide (ITO), are undoubtedly the most predominant materials in the field of optoelectronic applications due to their satisfactory transmittance (> 90%) and sheet resistance (10–25 Ω/□).^[^ ^13^ ^]^ However, the inherent properties of ITO such as high brittleness and poor flexibility hinder its applications for the emergent wearable electronics^[^ ^14^, ^15^ ^]^ and personal healthcare monitoring devices in which mechanical flexibility and stretchability are demanded.^[^ ^16^ ^]^ Alternative materials to ITO have been actively explored,^[^ ^17^ ^]^ such as conductive polymers,^[^ ^18^, ^19^ ^]^ carbon‐based materials (carbon nanotubes,^[^ ^20^, ^21^, ^22^ ^]^ graphene,^[^ ^7^, ^23^ ^]^ etc.), and metal‐based materials.^[^ ^24^ ^]^ Though high flexibility and stretchability could be achieved, a common shortage of conductive polymers and carbon‐based materials is their inferior electrical conductivity. On the other hand, metal‐based materials have attracted more interest recently because of their intrinsically high electrical conductivity, flexibility, and stretchability. To realize high transparency for the metal‐based TCEs, three types of structures, including ultrathin metal films,^[^ ^25^, ^26^ ^]^ metal nanowire networks,^[^ ^27^ ^]^ and metal meshes,^[^ ^28^, ^29^, ^30^ ^]^ are employed and extensively studied. While it is difficult to achieve high transparency and conductivity simultaneously for ultrathin metal films and metal nanowires, the trade‐off between the transparency and conductivity could be balanced by metal meshes via tuning their geometric parameters, such as line width, pitch, and thickness.^[^ ^28^ ^]^ As a consequence, metal meshes emerge as the most promising candidates for flexible and stretchable TCEs.

So far, two distinct approaches have been employed to fabricate metal meshes, namely conventional micro/nano fabrication process and solution‐based process.^[^ ^31^ ^]^ In the conventional micro/nano fabrication process, metal meshes are obtained by a combination of various techniques, including patterning technique (photolithography,^[^ ^32^ ^]^ electron beam (E‐beam) lithography,^[^ ^33^ ^]^ or nanoimprint lithography^[^ ^34^, ^35^ ^]^) to define mesh geometries, vacuum‐based film deposition technique (thermal evaporation,^[^ ^34^ ^]^ E‐beam evaporation, or sputtering^[^ ^36^ ^]^) to deposit uniform metal films, and wet etching^[^ ^32^ ^]^/lift‐off^[^ ^37^ ^]^ technique to transfer the defined patterns. Despite of the achievable high resolution (tens of nanometers for line width),^[^ ^33^, ^34^ ^]^ the conventional micro/nano fabrication process is often deemed to be a high‐cost and capital‐intensive process because of its subtractive manufacturing nature as well as the need of the vacuum‐based film deposition technique. In addition, the high vacuum film deposition technique is incompatible with flexible substrates (usually porous with lots of gas traps), limiting the application of the conventional micro/nano fabrication process in the popular wearable electronics. On the contrary, the solution‐based process has been drawing increasing attentions recently owing to its properties of additive manufacturing, low cost, high throughput, and high scalability. Printing technique is a typical representation of the solution‐based process.^[^ ^38^, ^39^ ^]^ However, the frequently used printing techniques, such as inkjet printing,^[^ ^40^, ^41^, ^42^ ^]^ gravure printing,^[^ ^43^ ^]^ offset printing,^[^ ^44^ ^]^ flexographic printing,^[^ ^45^, ^46^ ^]^ and screen printing,^[^ ^47^ ^]^ have limited resolution of tens of micrometers. Though a high resolution of tens of nanometers could be achieved by electrohydrodynamic (EHD) printing process,^[^ ^48^, ^49^, ^50^ ^]^ the EHD printing is inherently a serial process, facing scalability issues. Likewise, laser direct writing which possesses a serial manufacturing nature also faces scalability issues.^[^ ^51^, ^52^, ^53^ ^]^ In order to realize high resolution and high scalability simultaneously, several template‐assisted solution‐based fabrication processes have been developed. As an example, when conducting electroplating in E‐beam lithography created patterns, copper meshes with line width in sub‐micro scale (≈400 nm) can be fabricated in a few minutes,^[^ ^54^ ^]^ regardless of the substrate size, which is much faster than the EHD printing and laser writing processes. One problem of the electroplating process is that conductive substrates are required, which limits the practical application of the fabricated mesh structures. Although this problem could be solved by applying a transfer printing step, the extra step increases the complexity of the mesh fabrication process. A simpler way to fabricate metal meshes is to squeeze or blade nanoparticle inks directly into pre‐prepared micro/nano patterns.^[^ ^55^, ^56^ ^]^ However, nanoparticles may reside in the empty spaces (non‐patterned regions),^[^ ^55^ ^]^ which seriously deteriorates the transparency of the fabricated meshes. The issue of poor selectivity could be overcome by directed assembly process^[^ ^57^ ^]^ in which nanoparticles are precisely driven to desired regions under various external fields, such as electric field^[^ ^58^ ^]^ and fluidic flow.^[^ ^59^ ^]^ Fluidic flow‐directed assembly (also called as capillary assembly), which uses solvent evaporation‐induced convective flow to drive particle assembly, has been used to fabricate fine silver meshes with line width down to 150 nm.^[^ ^60^, ^61^ ^]^ However, a low assembly speed of < 220 µm^−1^ s is usually required in order to prolong the evaporation time and induce sufficient fluidic flow, which sets up an enormous obstacle to the application of the fluidic assembly process. Therefore, the development of a directed assembly process with enhanced efficiency is highly desirable.

Here, we introduce a surface energy‐directed assembly (SEDA) process to efficiently fabricate high resolution (line width down to 2 µm) silver meshes. The SEDA process combines solution coating methods, such as dip coating, blade coating, or spin coating, with a substrate wettability patterning strategy, wherein the function of the coating methods is to deposit a uniform nanoparticle suspension film on the substrate, while the function of the wettability patterning is to site‐selectively confine the nanoparticle suspension onto hydrophilic pattern regions. The confined suspension will then dry and leave behind nanoparticles therein, forming micro/nano structures that resemble the geometry of the hydrophilic patterns. The necessity of using a two‐step SEDA process rather than a one‐step SEDA process is demonstrated. The thickness of the assembled silver meshes can be tuned by varying assembly parameters. The assembled silver meshes exhibit excellent optoelectronic properties as well as excellent mechanical stability, and their applications in touch screen panels (TSPs) and thermal heaters (THs) are demonstrated.

## 2 Results and Discussions

### 2.1 Patterned Substrate Preparation and the Two‐Step SEDA Process

**Figure 1:**
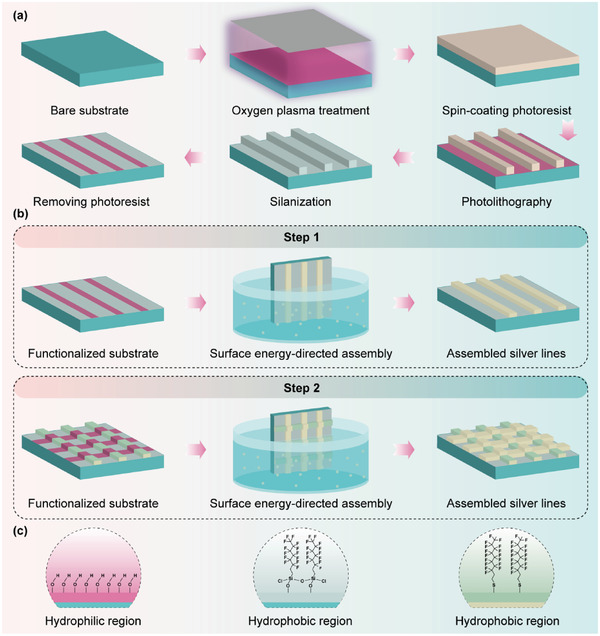
a) Schematic illustration of the process of the functionalized substrate preparation. b) Schematic illustration of the two‐step SEDA process for the fabrication of the silver mesh TCEs. The silver mesh TCEs are decomposed into two layers of stripe patterns, orthogonal to each other. During each SEDA step, silver nanoparticles are assembled on one layer of stripes. c) Chemical groups on the functionalized substrate: hydroxyl (‐OH) groups for the hydrophilic region (left), PFOCTS SAM for the hydrophobic region (middle), and PFDT SAM on the surface of the assembled silver nanoparticles for the hydrophobic region (right).

The SEDA process starts with the fabrication of a functionalized substrate with wettability contrast patterning (**Figure** **1**a). The substrate is first treated with oxygen (O_2_) plasma to render the surface hydrophilic. Negative tone photoresist or E‐beam resist is spin coated on the substrate and patterned using photolithography or E‐beam lithography to create micro or nano scale patterns. Then, the patterned substrate is treated with O_2_ plasma again, followed by chemical vapor deposition of a self‐assembly monolayer (SAM) film like trichloro (1H, 1H, 2H, 2H‐trifluorooctyl) silane (PFOCTS) which makes the substrate surface hydrophobic except for the resist protected regions. After the SAM film deposition, the substrate is immersed in a solvent such as acetone or dimethyl sulfoxide and then sonicated to remove the photoresist or E‐beam resist, exposing the resist protected hydrophilic patterns and resulting in a functionalized substrate surface with patterned wettability. It should be noted that the functionalized substrate could be fabricated using more efficient fashions like microcontact printing of patterned SAMs.^[^ ^62^ ^]^

**Figure 2:**
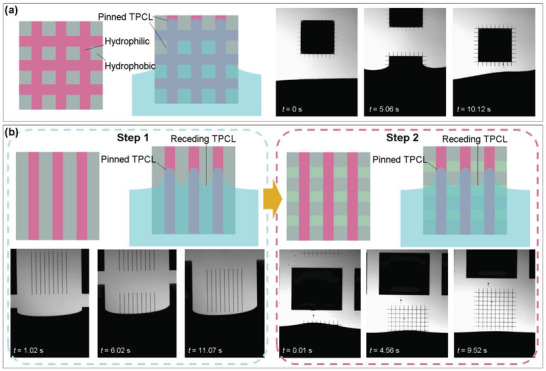
Fabrication of silver meshes using one‐step and two‐step SEDA processes. a) During the one‐step SEDA process, the TPCL pins on the hydrophilic mesh outline, resulting in covering of the silver nanoparticle suspension on the entire mesh outline‐enclosed region and the absence of assembly selectivity. b) The two‐step SEDA process turns from assembly on the mesh pattern into assembly on stripe patterns. As the TPCL pins on the stripe patterns while recedes freely at the non‐pattern regions in between, assembly could be implemented with excellent selectivity.

During the SEDA process, the functionalized substrate is dipped into a nanoparticle suspension and then withdrawn rapidly at a fixed speed in the range of 0.1 to 6 mm^−1^ s. The high pulling speed causes entraining of the nanoparticle suspension solely onto the hydrophilic pattern regions, realizing site‐selective assembly of nanoparticles into micro/nano structures after drying of the entrained suspension. A two‐step SEDA process is utilized in this paper for the fabrication of silver mesh TCEs (Figure 1b). Prior to discussing why the two‐step SEDA process is utilized, the fabrication of the silver mesh TCEs using a one‐step SEDA process is exhibited. During the assembly process, a functionalized substrate with hydrophilic mesh pattern is employed, as shown in **Figure** **2**a. The detailed assembly process is recorded by a high‐speed black‐and‐white camera (see Video S1, Supporting Information), and snapshots showing typical assembly stages are displayed in Figure 2a. Unexpectedly, a layer of nanoparticle suspension covers the entire mesh outline‐enclosed region, including hydrophobic areas between the mesh lines. After drying of the suspension, the whole region is covered by silver nanoparticles, meaning absent of assembly selectivity. The two‐step SEDA process is therefore developed to solve the selectivity issue. In the two‐step SEDA process, the silver mesh is decomposed into two layers of stripe patterns, orthogonal to each other. For each SEDA step, silver nanoparticles are assembled on one layer of stripes, turning from assembly on the mesh pattern into assembly on the stripe pattern. After successively assembling on the two layers of stripes and rotating the substrate 90^o^ in between, silver mesh TCEs are successfully fabricated. It can be seen from the recorded video (Video S2, Supporting Information) and snapshots (Figure 2b) that each step of the assembly process is accomplished with excellent selectivity and the assembly behaviors are almost identical for the two steps.

### 2.2 Mechanism of the Two‐Step SEDA Process

**Figure 3:**
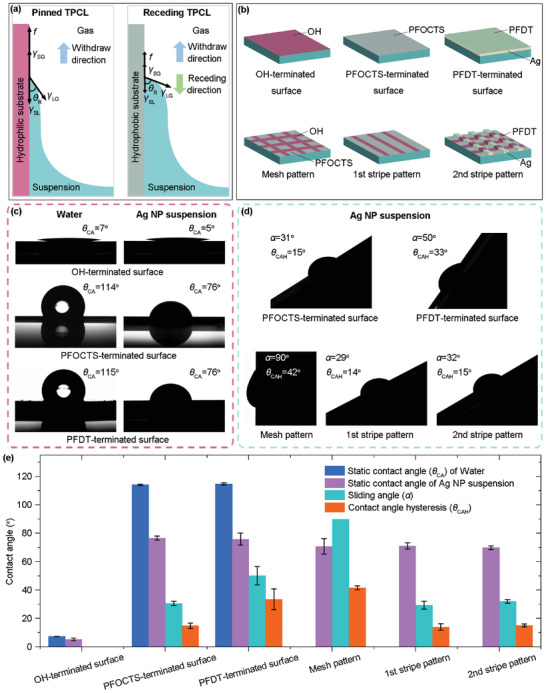
a) Force balance at the TPCL for hydrophilic and hydrophobic substrates. b) Schematic illustrations of six functionalized surfaces involved in this work. c) Static contact angles of water and silver (Ag) nanoparticle (NP) suspension droplets (20 µL) on OH‐terminated, PFOCTS‐terminated, and PFDT‐terminated surfaces. d) Sliding of Ag NP suspension droplets (20 µL) on PFOCTS‐terminated surface, PFDT‐terminated surface, mesh pattern, 1st stripe pattern and 2nd stripe pattern. e) Static contact angles, sliding angles, and contact angle hysteresis of various functionalized surfaces.

Prior to explaining the reason why the assembly selectivity for the stripe pattern is superior to that of the mesh pattern, the dynamics of the solid‐liquid‐gas three‐phase contact line (TPCL) is first analyzed (**Figure** **3**a). The force balance at the TPCL can be described with a modified Young's equation:^[^ ^63^, ^64^ ^]^

(1) $$\gamma_{\mathrm{SG}}+f\;=\gamma_{\mathrm{SL}}\;+\gamma_{\mathrm{LG}}\cos\theta_{R}$$

where *γ*_SG_ is the surface energy of the substrate, *γ*_LG_ is the surface tension of the suspension, *γ*_SL_ is the interfacial tension of the substrate‐suspension interface, *θ*_R_ is the contact angle of the suspension on the substrate, and *f* is the friction force per unit length exerted by the substrate to the TPCL. The terms on the left‐hand side of Equation 1 determine whether the TPCL pinning or receding on the substrate. For the hydrophilic substrate, the surface energy is always high, and the reachable friction force (maximum static friction force) is large because of the polar surface groups, facilitating pinning of the TPCL (Figure 3a). While for the hydrophobic substate with non‐polar surface groups, the surface energy is low, and the maximum friction force the substate can apply to the TPCL is small. Therefore, the TPCL is prone to recede. The receding of the TPCL on the hydrophobic substrate can be demonstrated by the droplet sliding experiment. A droplet of the silver nanoparticle suspension with a volume of 20 µL is injected onto the substrates. Then the stage starts to rotate, and the rotating angle of the stage is monitored. The rotating angle at which the droplet begins to slide on the substrates is the sliding angle. Advancing and receding contact angles are recorded at the sliding angle. The difference between the advancing contact angle and the receding contact angle is the contact angle hysteresis. For the PFOCTS‐terminated hydrophobic substrate (defined in Figure 3b) with static contact angles of 114^o^ for water and 76^o^ for the silver nanoparticle suspension (Figure 3c), the silver nanoparticle suspension droplet starts moving at a sliding angle of 31^o^ (Figure 3d and Video S3). Meanwhile, the contact angle hysteresis is only 15^o^, meaning easy receding of the TPCL.

During the SEDA process on the stripe pattern, as the stripe width to pitch ratio is 1:20, the proportion of the hydrophilic stripes at the TPCLs is only 4.76%. Even if the TPCL is pinned locally at the hydrophilic stripes, the main TPCL can still recede freely at the hydrophobic regions in between (Figure 2b), explaining why excellent assembly selectivity could be achieved. However, when conducting the SEDA process on the mesh pattern, the receding of the TPCL is prohibited by the hydrophilic mesh outline (Figure 2a). As a consequence, the entire mesh outline‐enclosed region is covered by the suspension and site‐selective assembly behavior of the SEDA process vanishes. More details about why the receding of the TPCL is prohibited by the hydrophilic mesh outline can be found in the Supporting Information, pp 1–2. The ability of TPCL receding on the stripe pattern and the disability of TPCL receding on the mesh pattern could also be demonstrated by the droplet sliding experiments (Figure 3d and Video S3, Supporting Information). For the stripe pattern, a continuous sliding behavior is observed. The sliding angle and the contact angle hysteresis are both small, which are close to those of the PFOCTS‐terminated hydrophobic substrate and are 29° and 14°, respectively. However, for the mesh pattern, even though the substrate turns 90° from the horizontal orientation, the droplet could not fall off. As described above, in the two‐step SEDA process, assembly is done on the stripe pattern in fact, rather than the mesh pattern. Before each SEDA step, the substrate is functionalized to make the stripe pattern regions hydrophilic and the regions between stripes hydrophobic. Therefore, the assembly process can be realized with excellent selectivity. A key challenge of the two‐step SEDA process is cross‐contamination caused by delamination of nanoparticles assembled in the first step or over‐assembly of nanoparticles on the as‐assembled micro/nano structures. Fortunately, the cross‐contamination issue is not encountered in this work, as detailed in the Figure S3 (Supporting Information, pp 2–3). This is because the formulation of the nanoparticle suspension is well optimized with excellent adhesion force with the substrate. In addition, the substrate is well functionalized to possess high hydrophilic/hydrophobic contrast. For the substrate functionalization, 1H, 1H, 2H, 2H‐perfluorodecanethiol (PFDT), a thiol‐based SAM, is utilized in addition to the silane‐based PFOCTS SAM in order to react with the assembled silver nanoparticles and render their surfaces hydrophobic (Figure 1c and Figure 3c). The sliding angle and the contact angle hysteresis for the droplet on the PFDT‐terminated silver surface are 50° and 33°, respectively, demonstrating that the droplet can fall off and the TPCL can recede freely.

To demonstrate the necessity and applicability of the two‐step SEDA process, one‐step SEDA experiments using various pure solvents have been conducted for the mesh pattern. The selected solvents as well as their surface tensions and viscosities are listed in **Table** **1**. All the solvents are alcohol ether‐based, which is the same as the silver nanoparticle suspension. As we expected, for all the solvents, a layer of solvent covers the entire mesh outline‐enclosed region (Figure S4 and Video S5, Supporting Information), meaning absent of assembly selectivity. If nanoparticles are dispersed in the solvents, the whole mesh outline‐enclosed region will be covered by the nanoparticles after the solvent is dried. This phenomenon is the same to what we observed during the assembly of the silver nanoparticle suspension. To achieve good assembly selectivity, the two‐step SEDA process proposed in this work should be utilized.

**Table 1:** Selected solvents as well as their surface tensions and viscosities.

Solvents	Surface tension (mN/m)	Viscosity (mPa.s)
Silver nanoparticle suspension	32.07	10.12
Triethylene glycol momobutyl ether	32.15	12.42
Diethylene glycol monomethyl ether	32.36	6.41
Ethylene glycol isopropyl ether	33.44	4.38
Ethylene glycol monomethyl ether	25.37	2.82
Ethylene glycol monophenyl ether	37.76	37.66
Diethylene glycol	43.92	39.47

### 2.3 Control of the SEDA Process

**Figure 4:**
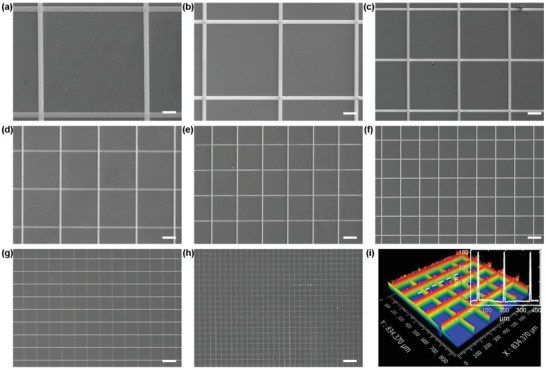
SEM images of silver meshes with a line width and line pitch ratio of 1:20. a) Line width 40 µm. b) Line width 30 µm. c) Line width 20 µm. d) Line width 15 µm. e) Line width 10 µm. f) Line width 7.5 µm. g) Line width 5 µm. h) Line width 2 µm. Scale bar in a‐h: 100 µm. i) 3D morphology of the silver mesh with a line width of 5 µm and a line pitch of 100 µm. Inset is a cross‐sectional profile along the white dashed line.

Using the two‐step SEDA process, silver meshes with line widths from 40 to 2 µm are fabricated, as shown in **Figure** **4**a–h. The line pitch increases with the line width at a ratio of 20:1 in order to maintain a constant fill factor of ≈0.1. The fill factor represents the coverage ratio of the mesh pattern on the substrate and can be calculated by:^[^ ^10^ ^]^

(2) $$\;f_{F}=\;1-\frac{{\left(p-w\right)}^{2}}{p^{2}}$$

where *p* and *w* stand for line pitch and width, respectively. Therefore, a transmittance of ≈90% could be calculated for all the fabricated silver meshes, given by:^[^ ^10^ ^]^

(3) $$T\;=\;1-f_{F}$$

The 3D morphology of the silver mesh with a line width of 5 µm and a line pitch of 100 µm measured by a white light interference microscope is shown in Figure 4i. It can be seen from the micrograph that metal mesh with a uniform line thickness is fabricated. The sheet resistance of the silver meshes can be calculated by:^[^ ^10^ ^]^

(4) $$\;R_{s}=\frac{\rho }{f_{F}}\;\frac{1}{h}$$

where *ρ* is the resistivity of silver, and *h* is the line thickness. As all the silver meshes possess the same fill factor, the line thickness becomes the determining factor for the sheet resistance.

**Figure 5:**
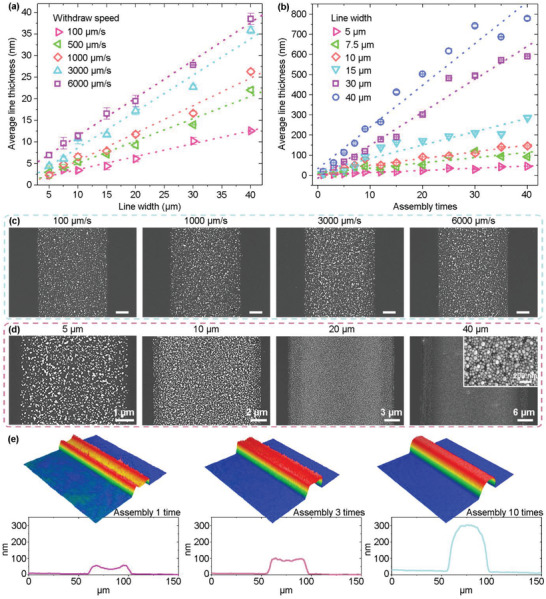
Controlling of the average line thicknesses. a) Average line thickness as a function of line width. b) Average line thickness as a function of assembly times. c) SEM images of silver lines with a line width of 5 µm assembled at various withdraw speeds. Scale bar: 1 µm. d) SEM images of silver lines with line widths from 5 µm to 40 µm assembled at a constant withdraw speed of 3000 µm^−1^ s. e) 3D morphologies and cross‐sectional profiles of silver lines with a width of 40 µm assembled for different times.

The line thickness of the assembled silver meshes depends on the line width (equivalent to the width of the hydrophilic stripes) and the withdraw speed, as exhibited in **Figure** **5**a and Figure S5 (Supporting Information). The influence of the line width and the withdraw speed on the line thickness can be understood from the modified Landau‐Levich‐Derjaguin (LLD) theory proposed by Darhuber et al.^[^ ^65^ ^]^ The theory predicts the thickness of the entrained suspension on the hydrophilic stripes during dip coating on functionalized substrates using the following equation:

(5) $$t\;=\;0.356w{\left(\frac{\mu V}{\gamma }\right)}^{1/3}$$

where *µ* and *γ* are the dynamic viscosity and surface tension of the suspension, respectively, and *V* is the withdraw speed. According to Equation 5, the entrained suspension thickness *t* is proportional to the line width *w* and the 1/3 power of the withdraw speed *V*. The increase of the thickness of the entrained suspension with the withdraw speed could be clearly seen in Figure S6 (Supporting Information). Because the concentration of silver nanoparticles in the suspension is fixed and the nanoparticles are distributed uniformly in the suspension, the line thickness of the assembled silver meshes should be proportional to the thickness of the entrained suspension, and therefore should also be proportional to the line width and the 1/3 power of the withdraw speed. It can be seen from the experimental data and the fitted data obtained using Equation 5 that the line thickness does increase linearly with the line width (Figure 5a). In addition, the line thickness increases according to the 1/3 power of the withdraw speed, as exhibited in Figure S5 (Supporting Information). It should be noted that when adjusting the line width and the withdraw speed, the obtained line thickness is only in the range from 2 to 38 nm, which is even smaller than the diameters (30–50 nm) of the silver nanoparticles. The reason lies in that the volume of the nanoparticle suspension entrained on the patterned region is small. The small volume of suspension can only contain limited number of nanoparticles, leaving behind deficient nanoparticles after the suspension is dried. As can be seen from Figure 5c,d, only a small number of nanoparticles are assembled at a line width of 5 µm and a withdraw speed of 100 µm ^−1^s. These nanoparticles populate sparsely on the patten regions, leading to a small average thickness of only a few nanometers. At a large line width of 40 µm and a high withdraw speed of 3000 µm^−1^ s, the assembled nanoparticles become denser. However, voids still exist in the assembled lines, again resulting in the small average thickness. More SEM images of silver lines with different widths assembled at various withdraw speeds can be found in Figure S7 (Supporting Information). The sparse population of nanoparticles can definitely affect the resistance of the assembled silver lines. In order to get a larger line thickness, suspensions with a higher nanoparticle concentration should be used. However, this is unlikely to be achievable as the nanoparticle concentration (25–30 wt.%) is already pretty high and a higher concentration may affect the stability of the suspension. Another way to increase the line thickness is doing assembly for multiple times. As can be seen in Figure 5b, the line thickness increases almost linearly with the number of the assembly times. For the multiple time assembly process, it is crucial to have a time interval between adjacent assembly steps for the entrained suspension to be dried. Otherwise, the entrained suspension will flow back to the bulk suspension when the substrate is dipped in. At a line width of 40 µm, a minimum time interval of 200 s is required (Figure S8, Supporting Information). For thinner lines with a line width of 7.5 µm, as the volume of the entrained suspension is smaller, the time interval could be shortened to < 20 s (Figure S8, Supporting Information). In this paper, the time interval for the multiple time assembly process is fixed at 200 s. Besides increasing the line thickness, the multiple time assembly process can help suppress the well‐known “coffee‐ring” effect^[^ ^66^ ^]^ which is commonly encountered during drying of a liquid droplet. The detailed reason can be found in the Supporting Information, pp 7–8. For the silver line assembled for only one time, a “coffee‐ring” pattern with thick edges is formed, as exhibited in Figure 5e. However, when increasing the assembly times, the “coffee‐ring” pattern disappears (Figure 5e and Figure S9, Supporting Information).

### 2.4 Electrical and Optical Properties of the Assembled Silver Meshes

**Figure 6:**
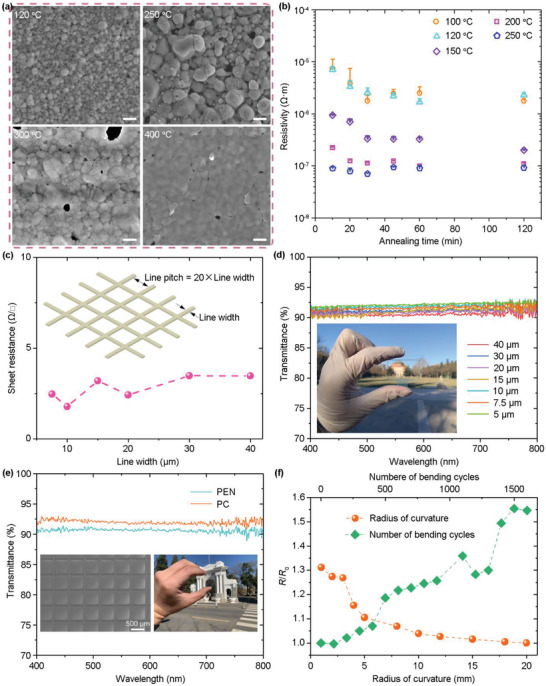
Sheet resistance, optical transmittance and mechanical stability of silver meshes fabricated on silicon, glass, PC and PEN substrates. a) SEM images of the assembled nanoparticles under different annealing temperatures. Scale bar: 200 nm. b) Resistivities of silver patterns as a function of the annealing temperature under annealing time between 10 to 120 min. c) Sheet resistances of silver meshes with different line widths and the same line thickness (≈400 nm) fabricated on glass substrates. d) Optical transmittance spectra of silver meshes with different line widths fabricated on glass substrates. The inset is a photograph of the silver mesh. e) Optical transmittance spectra of the silver meshes fabricated on PEN and PC substrates. Insets are a SEM image and a photograph of a silver mesh fabricated on a PEN substrate. f) Sheet resistance stability of the silver mesh fabricated on the PEN substrate over 1500 bending cycles with different radii of curvature.

After assembly of silver meshes, a thermal annealing step is always required to sinter and merge nanoparticles in order to get a high conductivity. To investigate the sintering property of the silver nanoparticles, the SEDA process is done on patterns 40 µm by 100 µm in size at a withdraw speed of 3000 µm^−1^ s for 4 assembly times. The resulting thickness of the silver patterns is ≈150 nm. **Figure** **6**a shows SEM images of assembled nanoparticles annealed under various temperatures. At a low annealing temperature of 120 °C, coalescence of nanoparticles does not happen, and isolated nanoparticles could be clearly seen. With the increase of the annealing temperature, coalescence of nanoparticles happens, and sintered films are formed. The resistivities of annealed silver patterns are measured using four‐probe method (as detailed in the Figure S10, Supporting Information), and the results are plotted in Figure 6b. As the annealing temperature increases from 100 to 250 °C, the resistivity of the silver pattern decreases by almost two orders of magnitude, attributing to the coalescence of nanoparticles. A lowest resistivity of 7.13 × 10^−8^ Ω m is achieved at an annealing temperature of 250 °C, which is only 4.5 times higher than the bulk resistivity of silver (1.59 × 10^−8^ Ω m). One possible reason for the slightly high resistivity is the presence of voids in the assembled silver pattern. The high annealing temperature of 250 °C is not compatible with flexible substrates which typically can only resist an annealing temperature of below 200 °C. Fortunately, comparable resistivities could be achieved at an annealing temperature of 200 °C by extending the annealing time to > 60 min. Therefore, an annealing temperature of 200 °C and an annealing time of 60 min are utilized hereafter.

Figure 6c shows sheet resistances of silver meshes with different line widths and the same line thickness (≈400 nm) fabricated on glass substrates. As the line thickness and the fill factor keep the same, according to Equation 4, the sheet resistances should be equal for all the meshes. This deduction is demonstrated by the almost constant resistance data in Figure 6c. A lowest sheet resistance of 1.79 Ω/□ is obtained for the silver mesh with a line width of 10 µm. Figure 6d shows optical transmittance spectra of the fabricated silver meshes. The silver meshes are highly transparent in the visible light regime with transmittances in the range of 90.5 to 92% at a wavelength of 550 nm. Inset in Figure 6d is a photograph of a transparent silver mesh.

Besides rigid substrates, the two‐step SEDA process is applicable to flexible substrates such as polyethylene naphthalate (PEN) and polycarbonate (PC) as well. Figure 6e shows optical transmittance spectra of the silver meshes with a line width of 10 µm fabricated on PEN and PC substrates. Similar to glass substrates, the transmittances of the silver meshes on PEN and PC substrates are both above 90%, demonstrating a high transparency. Insets in Figure 6e are a SEM image and a photograph of a silver mesh fabricated on a PEN substrate. The silver mesh has a low sheet resistance of 16.4 Ω/□. In addition, the silver mesh exhibits a remarkable mechanical stability, as demonstrated by the cyclic bending results in Figure 6f. After over 1500 bending cycles with different radii of curvature between 1 and 20 mm, the sheet resistance increases less than 1.6 times, indicating good adhesion force between the silver nanoparticles with the substrates.

### 2.5 Process Benchmark

**Figure 7:**
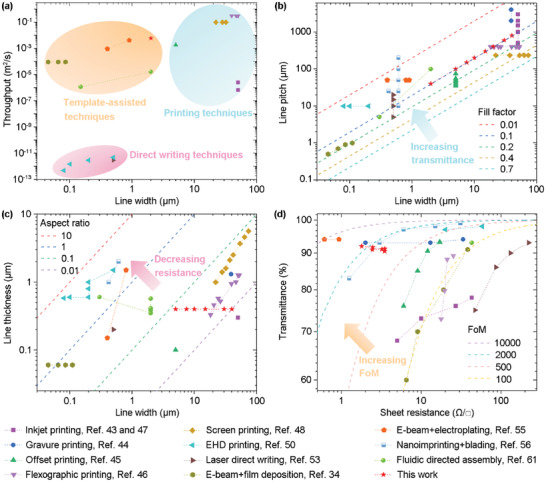
Comparation of the line width, line pitch, line thickness, fabrication efficiency, sheet resistance, and optical transmittance achieved in this work with those from literatures. a) Line width versus fabrication efficiency from this work and literatures. b) Line width versus line pitch from this work and literatures. The dashed lines fit with iso‐values of the fill factor: 0.01, 0.1, 0.2, 0.4, and 0.7. c) Line width versus line thickness from this work and literatures. The dashed lines fit with iso‐values of the aspect ratio: 0.01, 0.1, 1 and 10. d) Optical transmittance versus sheet resistance for metal meshes from this work and others literatures. The dashed lines fit with iso‐values of the FoM: 100, 500, 2000, and 10 000.

The line width, line pitch, line thickness, fabrication efficiency, sheet resistance, and optical transmittance achieved in this work are compared with those from literatures, as plotted in **Figure** **7**. A line width down to 2 µm is achieved in this work, which is superior to those achieved by the printing techniques (such as inkjet printing, offset printing, flexographic printing, and screen printing) but inferior to those achieved by EHD printing and laser writing (Figure 7a). However, the fabrication efficiency in this work is inferior to the former and superior to the latter. Therefore, in terms of overall performance, the SEDA process in this work outperforms most of the previous processes. In addition, it should be mentioned that, as one of the template‐assisted fabrication processes, when substrates with nanoscale hydrophilic patterns are fabricated and utilized, the resolution of the SEDA process can be increased to nanoscale. Furthermore, compared with the conventional micro/nano fabrication process, the SEDA process does not need vacuum and are additive in nature. Therefore, the fabrication efficiency of the SEDA process should be significantly higher and the cost should be lower than the conventional micro/nano fabrication process. A detailed comparation of the efficiency of the two‐step SEDA process and the conventional micro/nano fabrication process could be found in the Supporting Information, pp 10–12. Compared with the electroplating process, conductive substrates are not required in the SEDA process. Compared with the fluidic directed assembly process, the fabrication efficiency of the SEDA process is significantly higher. In conclusion, with high resolution, high throughput, high versatility and additive nature, the SEDA process opens remarkable opportunities for the fabrication of metal meshes and other micro/nano structures.

According to Equation 2, the line width and line pitch determine the transparency of the metal meshes. In this work, the line width and line pitch vary at the same pace in order to maintain a constant fill factor of 0.1 which corresponds to a transmittance of 90%. In the other works, the line width and line pitch are arranged following a similar logic. The fill factors are mostly in the range of 0.01 to 0.2, corresponding to transmittances of 80%–99% (Figure 7b). With the same line width and line pitch, a larger line thickness results in a smaller sheet resistance, meaning that a higher thickness to width aspect ratio is expected. The highest aspect ratio achieved in this work is 0.08, which is comparable to those achieved by other works (Figure 7c).

The optical transmittance at a wavelength of 550 nm versus the sheet resistance for metal meshes in this work and in other literatures is displayed in Figure 7d. To compare the optoelectronic performance of these metal meshes, a figure of merit (FoM) value is calculated by:^[^ ^10^ ^]^

(6) $$\mathrm{FoM}\;=\frac{188.5}{R_{s}}\;\frac{1}{T_{550}^{-1/2}-1}$$

where *R*_s_ is the sheet resistance and *T*_550_ is the transmittance at 550 nm. The dashed lines in Figure 7d fit with iso‐values of the FoM: 100, 500, 2000, and 10 000. A high FoM value of 2465 is accomplished in this work, which is higher than most of the metal meshes fabricated using other techniques.

### 2.6 Applications of the Assembled Silver Meshes

**Figure 8:**
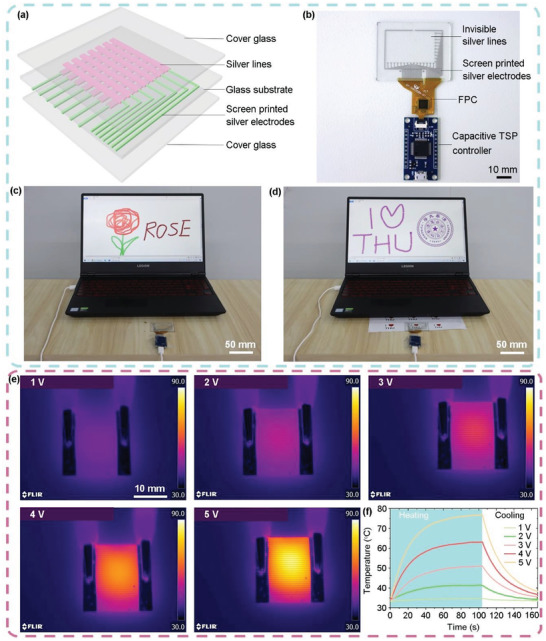
Practical application of the assembled silver meshes in TSPs and THs. a) Schematic illustration of the configuration of the capacitive‐type TSP. b) Photograph of the fabricated TSP device connected to a controller board. c) Demonstration of drawing a rose on the TSP using a stylus pen, which is displayed synchronously on the laptop monitor. d) Demonstration of writing characters “I ♥ THU”. Background shows the characters on a piece of paper, demonstrating the excellent transparency of the TSP. e) IR images of the TH heated up to various temperatures when applying DC voltages between 1 and 5 V. f) Time‐dependence of temperature when DC voltages between 1 and 5 V are applied to the TH.

The low sheet resistance of 1.79 Ω/□, high optical transmittance of ≈92%, and high FoM value of 2465 of the fabricated silver meshes demonstrate the possibility of replacing the widely used ITO film with sheet resistance of 10–25 Ω/□, optical transmittance of ≈90%, and FoM value of 140–350. To further demonstrate the potential of the assembled silver meshes for optoelectronic applications, a capacitive‐type TSP is fabricated, as schematically illustrated in **Figure** **8**a. Orthogonal silver lines with a width of 10 µm and a thickness of 150 nm are assembled on the front and back sides of a glass substrate (400 µm thick) successively. Detailed geometrical parameters of the silver lines can be found in Figure S11 (Supporting Information). The silver lines are connected to a controller of capacitive touch panel via screen printed electrodes. Then the glass substrate is sandwiched between two cover glasses, forming the TSP. Figure 8b shows a photographic image of the fabricated TSP device. The working mechanism of the capacitive‐type TSPs could be found in Ref.[5, 6, 67] By connecting the TSP to a laptop and employing the MS paint program, the TSP can be operated by a human finger or a stylus pen to draw pictures (Figure 8c, Video S6, Supporting Information) and write characters (Figure 8d, Video S7, Supporting Information). The writing and drawing could be done over the entire touch area without any disconnected lines or defects, demonstrating the outstanding uniformity and touch sensitivity of the TPS device. The uniform device performance can be further attributed to the excellent geometrical and electrical properties of the silver meshes.

Besides being used in TSPs, the assembled silver meshes could be utilized to construct flexible and transparent THs. THs are essentially resistors, using Joule heating to heat up themselves. They have lots of potential applications such as smart windows, deicers, window defrosters, *etc*.^[^ ^68^ ^]^ In this work, to fabricate a flexible TH, a silver mesh with a line width of 10 µm, a line pitch of 200 µm and a sheet resistance of 27 Ω/□ is assembled on a PEN substrate 20 by 20 mm in size. Then aluminum electrodes with a thickness of 200 nm are prepared at the two ends of the silver mesh. DC voltages between 1 and 5 V are supplied to the TH through the two aluminum electrodes, and the corresponding temperatures of the TH are monitored using a thermal infrared (IR) imaging camera, as shown in Figure 8e. With the increase of the DC voltage, the achievable temperature of the TH increases. The time‐dependent temperatures of the TH are measured under various DC voltages and are displayed in Figure 8f. The steady–state temperatures of the TH are reached within 40 s, and the temperatures drop rapidly (within 60 s) to the room temperature once the voltages are turned off. The time for the TH to reach steady‐state temperatures is the thermal response time, which is ≈40 s in this work. The thermal response time is independent of the applied voltage, which is consistent with the results reported previously.^[^ ^28^ ^]^ Figure S12 (Supporting Information) exhibits the stability of the fabricated TH during long‐term thermal cycles at an applied voltage of 3 V. No significant change in heating performance was observed after 21 thermal cycles, demonstrating the excellent stability.

## 3 Conclusion

In summary, we present a high resolution and high throughput surface energy‐directed assembly process which utilizes a functionalized substrate with patterned wettability to site‐selectively confine nanoparticles onto hydrophilic pattern regions, forming micro/nano structures. Experimental results and theoretical analysis indicate that pinning of the TPCL at the hydrophilic pattern regions determines the implementation of the assembly process while receding of the TPCL at the hydrophobic non‐pattern regions determines the assembly selectivity. For stripe patterns, the TPCL can recede freely, while for mesh patterns, the receding the TPCL is prohibited, resulting in absence of assembly selectivity. In order to achieve high assembly selectivity, a two‐step SEDA process is proposed in this work, which turns from assembly on mesh patterns into assembly on stripe patterns. Utilizing the two‐step SEDA process, silver meshes with a line width down to 2 µm are assembled on both rigid and flexible substrates. The thickness of the silver meshes could be tuned by varying the withdraw speed and the assembly times. The assembled silver meshes exhibit a low sheet resistance of 1.79 Ω/□ and a high optical transmittance of ≈92% (corresponding to a high FoM value of 2465) as well as an excellent mechanical stability. The potential applications of the silver meshes are demonstrated by a capacitive‐type TSPs and a TH. Having a lower fabrication cost compared with the conventional micro/nano fabrication process, a higher resolution compared with the printing techniques, and a higher efficiency compared with the EHD printing, laser writing and template‐assisted processes, the two‐step SEDA process in this work outperforms most of the previous fabrication processes in terms of overall performance, demonstrating the potential of using the two‐step SEDA process for the fabrication of TCEs for optoelectronic applications.

## 4 Experimental Section

### Functionalized Substrate Preparation

To prepare the functionalized substrate, a substrate was cleaned with oxygen (O_2_) plasma (100 W, 100 sccm, IoN Wave 10) for 3 min to render the surface hydrophilic with a contact angle of ≈5^o^. Negative photoresist (NR9‐3000PY, Futurrex) was spin coated on the substrate, exposed under an ultraviolet (UV) light for 40 s (URE‐2000), and developed for 1 min (RD6 developer, Futurrex) to create a patterned photoresist mask. The photoresist mask keeps the underlying substrate surface hydrophilic during the subsequent processing. The patterned substrate was cleaned using O_2_ plasma for 3 min again to remove residue photoresist. Then a silane‐based PFOCTS SAM film is deposited on the entire substrate using a chemical vapor deposition process by placing the substrate in a petri dish, adding 20 µL of trichloro (1H, 1H, 2H, 2H‐trifluorooctyl) silane (Aladdin^®^), and heating up the petri dish to 150 °C on a hotplate for 10 min. Post SAM deposition, the substrate was immersed in DMSO (Aladdin^®^) to remove the photoresist mask, exposing the photoresist covered hydrophilic patterns and resulting in a functionalized substrate with patterned wettability. The above procedure could be utilized for the functionalization of silicon and glass substrates. For PEN and PC substrates, a fluorine‐containing resin (PyFlon^TM^) film should be prepared prior to surface functionalization by a spin coating process to decrease their surface roughness.

When utilizing the two‐step SEDA process, the surface functionalization should be conducted twice. For the latter surface functionalization step, an argon (Ar) plasma should be used to clean the substrate surface instead of O_2_ plasma in order to avoid oxidation of the firstly assembled silver lines. In addition, a thiol‐based PFDT (1H, 1H, 2H, 2H‐perfluorodecanethiol, Aladdin^®^) SAM film is required to make the assembled silver lines hydrophobic.

### Materials

The silver nanoparticle suspension (BroadCON‐INK550) was purchased from BroadTeko Technology Co., Ltd. and used as it is. The average diameter of the silver nanoparticles is between 30 and 50 nm, and the concentration of the suspension is 25–30 wt.%. Silicon and glass (BF33) substrates were purchased from Top Vendor Science & Technology Co., Ltd. The PFOCTS and PFDT SAM solutions were purchased from Aladdin^®^.

### Fabrication of Silver Meshes using the SEDA Process

A two‐step SEDA process was utilized to fabricate silver meshes. During the first assembly step, a substrate was first functionalized to possess hydrophilic stripe patterns. The substrate was dipped vertically into a silver nanoparticle suspension parallel to the stripes, and then withdrawn at a fixed speed in the range of 0.1 to 6 mm^−1^ s using a dip coater (SYDC‐1, Sanyan Experimental Instrument Co., LTD). The silver nanoparticle suspension was site‐selectively entrained on the strip patterns, which formed silver lines after dried. After the first assembly step, the substrate was annealed on a hotplate at temperatures between 100 to 400 °C for a period of 10 to 120 mins in order to sinter assembled silver nanoparticles as well as enhance the adhesion between the nanoparticles and the substrate. The procedure was repeated again to conduct the second assembly step and finish the fabrication of the silver meshes.

### Fabrication of a Capacitive‐Type TSP

Orthogonal silver lines were assembled on the front and back sides of a glass substrate successively. Then the silver lines were connected to a capacitive TSP controller (NO2511P.C2.P1.V1) via screen printed electrodes and a flexible printed circuit (FPC). Finally, the glass substrate was sandwiched between two cover glasses using optical clear adhesive (OCA) films, forming the TSP. The TSP was operated by connecting the controller to a laptop via a universal serial bus (USB).

### Contact Angles

Static contact angles were measured using a contact angle measuring device (OCA25, Dataphysics). Solvent droplets with a volume of 20 µL were injected onto the substrates. Each contact angle value was an average of five measurements. The sliding angles and contact angle hysteresis were measured by the same tool. Each sliding angle value was an average of five measurements.

### Characterization of Silver Meshes

The movies of the assembly process was recorded by a contact angle measuring device (OCA25, Dataphysics) equipped with a coaxial light microscope. Microscope images of the silver meshes were taken employing an optical microscope (XJ‐52C, Puzhe Thotoelectric) and a scanning electron microscope (SU8220, Hitachi). The thickness and morphology were obtained by a white light interference microscope (Zygo Nexview) and an atomic force microscope (AFM, Bruker MultiMode 8). The transmittance of the silver meshes was measured by an ultraviolet‐visible spectrophotometer (Lambda 1050+, PerkinElmer). The resistivity and sheet resistance were measured by a probe station (TS2000‐SE, MPI) combined with a semiconductor parameter analyzer (B1500A, Keysight).

## Conflict of Interest

The authors declare no conflict of interest.

## Supporting information

Supporting Information

Supplemental Video 1

Supplemental Video 2

Supplemental Video 3

Supplemental Video 4

Supplemental Video 5

Supplemental Video 6

Supplemental Video 7

## Data Availability

The data that support the findings of this study are available from the corresponding author upon reasonable request.
