# Supplementary material for: Fabrication of Flexible and Transparent Metal Mesh Electrodes Using Surface Energy‐Directed Assembly Process for Touch Screen Panels and Heaters
Source: Adv Sci (Weinh). 2023 Oct 11;10(34):2304990. doi: 10.1002/advs.202304990 (PMC10700185; doi:10.1002/advs.202304990)
Supplement: Supplementary file 1 — Supporting Information [file ADVS-10-2304990-s004.pdf]

ADVANCED SCIENCE
Open Access

Supporting Information

for *Adv. Sci.*, DOI 10.1002/advs.202304990

Fabrication of Flexible and Transparent Metal Mesh Electrodes Using Surface Energy-Directed Assembly Process for Touch Screen Panels and Heaters

Siqing Yuan, Zebin Fan, Guangji Wang, Zhimin Chai, Tongqing Wang, Dewen Zhao, Ahmed A. Busnaina and Xinchun Lu*

Supporting Information

Fabrication of Flexible and Transparent Metal Mesh Electrodes Using Surface Energy-Directed Assembly Process for Touch Screen Panels and Heaters

Siqing Yuan,[1,2] Zebin Fan,[1,2] Guangji Wang,[1,2] Zhimin Chai,[1,2] Tongqing Wang,[1,2] Dewen Zhao,[1,2] Ahmed A. Busnaina,[3] and Xinchun Lu[1,2]*

[1]State Key Laboratory of Tribology in Advanced Equipment, Tsinghua University, Beijing 100084, China

[2]Department of Mechanical Engineering, Tsinghua University, Beijing 100084, China

[3]NSF Nanoscale Science and Engineering Center for High-Rate Nanomanufacturing (CHN), Northeastern University, Boston, Massachusetts 02115, United States

Corresponding Author: chaizhimin@mail.tsinghua.edu.cn

As shown in **Figure S1**b and c, excellent assembly selectivity could be achieved for the stripe patterns both parallel to and perpendicular to the moving TPCL. The reason lies in that the TPCL can recede freely at the hydrophobic regions. When conducting the SEDA process on the mesh pattern (Figure S1a), the receding of the TPCL in both vertical and horizontal directions is prohibited by the hydrophilic outline. As a consequence, the entire outline-enclosed region is covered by the nanoparticle suspension and site-selective assembly behavior of the SEDA process vanishes.

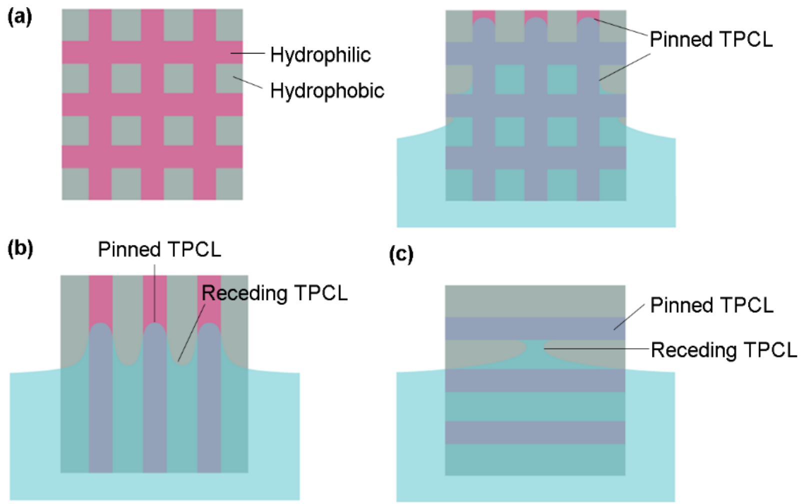

Figure S1. Schematic illustration of conducting the SEDA process on mesh and stripe patterns.

To further explain why the receding of the TPCL is prohibited for the mesh pattern, the assembly behavior of a square loop structure is investigated, as shown in **Figure S2** and **Video S4**. The square loop structure is an elementary unit of the mesh pattern. As we expected, the TPCL is pinned on the hydrophilic outline. Therefore, the receding of the TPCL is prohibited. The entire outline-enclosed region is covered by the nanoparticle suspension and site-selective assembly behavior of the SEDA process vanishes.

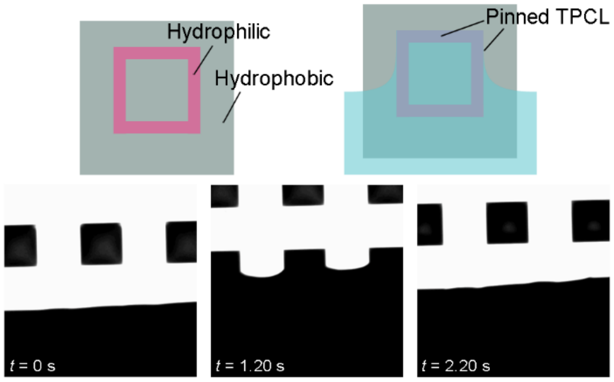

Figure S2. Fabrication of a square loop structure using the SEDA process.

To demonstrate that over-assembly of nanoparticles on the as-assembled silver lines does not exist in this work, the thickness of the 1st layer assembled silver line 7.5 μm in width before and after the 2nd step SEDA process is measured by atomic force microscope (AFM), as shown in **Figure S3**. As we see, after conducting the 2nd step SEDA process for 30 times,

the thickness for the 1st layer assembled silver line does not change much, meaning that no silver nanoparticles are lost or assembled in the 2nd step SEDA process.

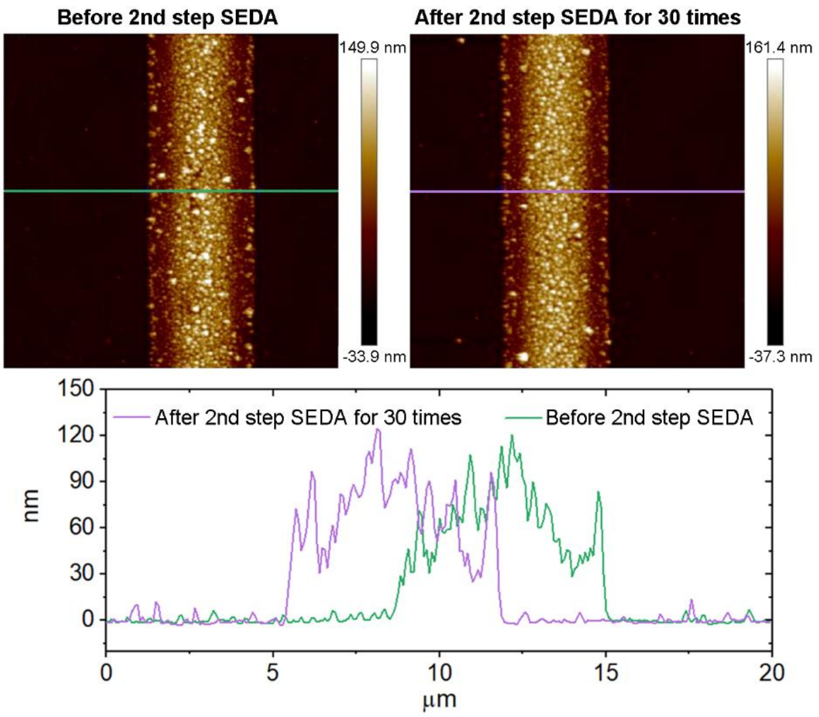

Figure S3. The thickness of the 1st layer assembled silver line 7.5 μm in width before and after the 2nd step SEDA process

To demonstrate the necessity and applicability of the two-step SEDA process, one-step SEDA experiments using various pure solvents have been conducted for the mesh pattern. The results are exhibited in **Figure S4**. For all the solvents, a layer of solvent covers the entire mesh outline-enclosed region, meaning absent of assembly selectivity. Breaking of the solvent film is observed for two solvents, ethylene glycol isopropyl ether and ethylene glycol monomethyl ether. The reason lies in that the boiling points of these two solvents are low, which are 144 ^{o}C and 124 ^{o}C, respectively. After the assembled solvents dry, the pinning of TPCL on the mesh outline disappears, resulting in receding of the TPCL and thus breaking of the solvent film. However, after adding 0.5 M zinc acetate dihydrate in the ethylene glycol monomethyl ether solvent, the TPCL can be pinned again because zinc acetate dihydrate becomes ZnO after dried.

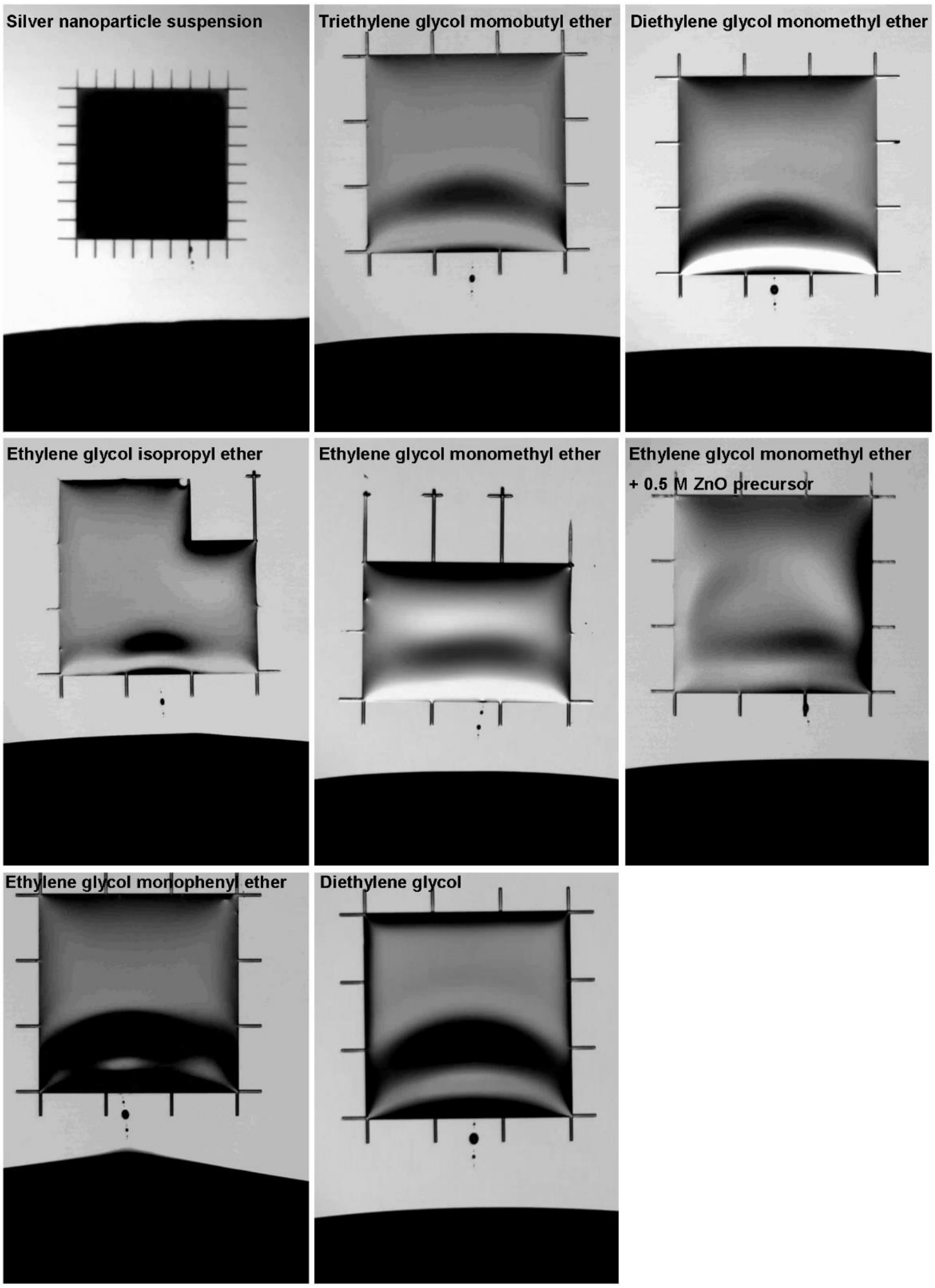

Figure S4. One-step SEDA for mesh patterns using various pure solvents

Figure S5 show the average line thickness of the assembled silver meshes as a function of the withdraw speed. The line thickness is proportional to the 1/3 power of the withdraw speed *V*.

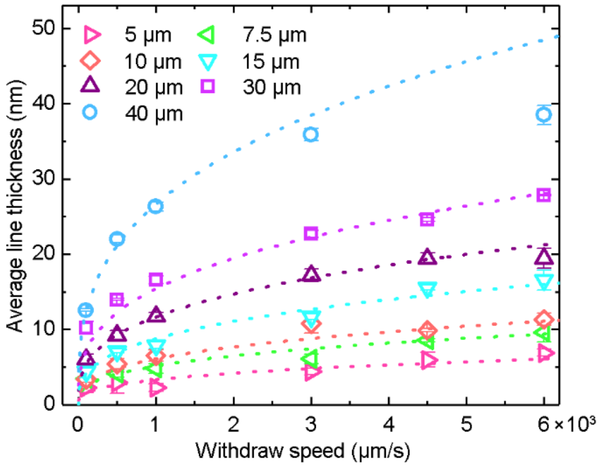

Figure S5. Average line thickness as a function of the withdraw speed.

The increase of the thickness of the entrained suspension with the withdraw speed could be clearly seen in **Figure S6**. When the withdraw speed increases from 100 μm/s (Figure S6a) to 1000 μm/s (Figure S6b), the color of the entrained suspension becomes darker, meaning increased thickness of the entrained suspension.

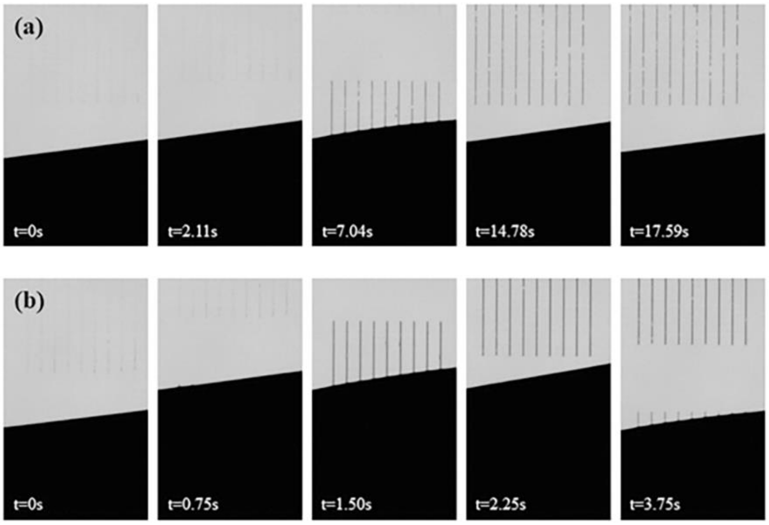

Figure S6. Fabrication of stripe patterns using the SEDA process at withdraw speeds of 100 μm/s (a) and 1000 μm/s (b).

Figure S7 shows SEM images of silver lines with different widths assembled at various withdraw speeds.

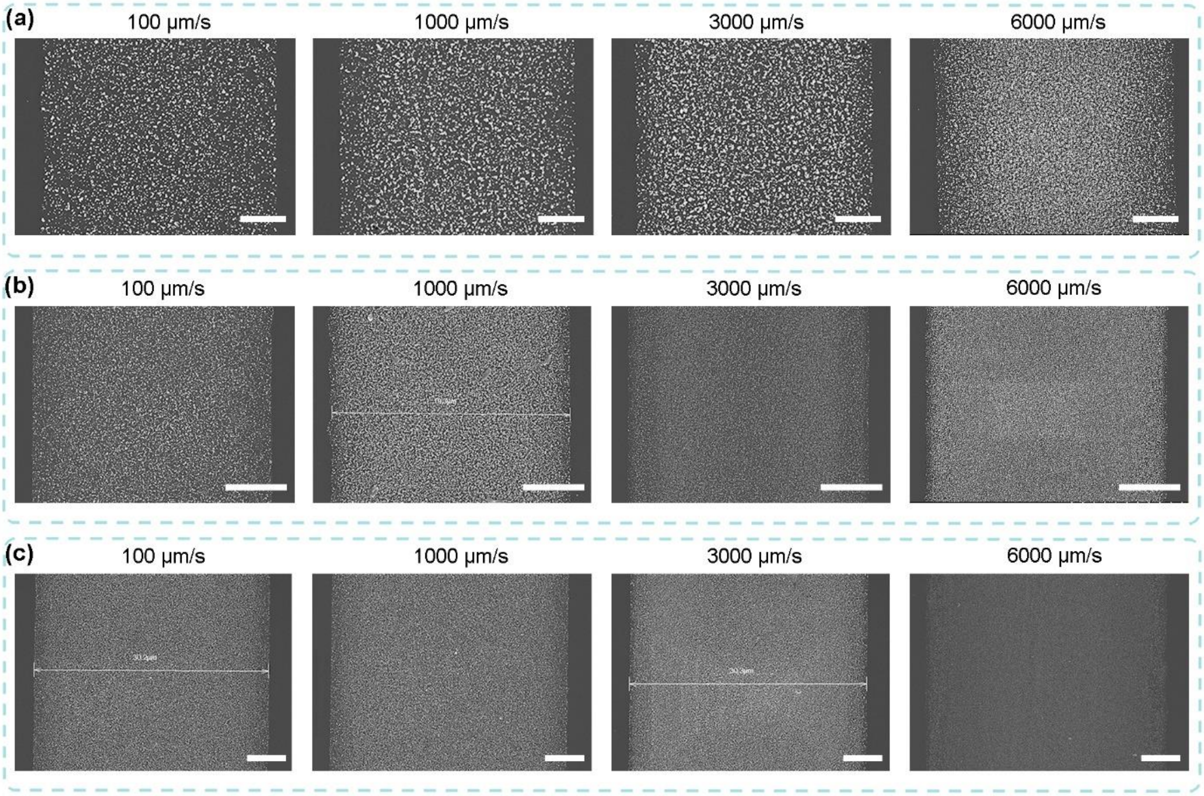

Figure S7. (a) SEM images of silver lines 10 µm in width assembled at various withdraw speeds. Scale bar: 2 µm. (b) SEM images of silver lines 20 µm in width assembled at various withdraw speeds. Scale bar: 5 µm. (c) SEM images of silver lines 30 µm in width assembled at various withdraw speeds. Scale bar: 5 µm.

For the multiple time assembly process, it is crucial to have a time interval between adjacent assembly steps for the entrained suspension to be dried. Otherwise, the entrained suspension will flow back to the bulk suspension when the substrate is dipped in, causing decreasing of the line thickness. At a line width of 40 µm, a minimum time interval of 200 s is required, as seen in **Figure S8**a. However, for thinner lines with a line width of 7.5 µm, as the volume of the entrained suspension is smaller, the time interval could be shortened to < 20 s (Figure S8b). In fact, we have demonstrated that for lines equal to or thinner than 7.5 µm, no time interval is needed because the entrained suspension dries immediately once the sample is pulled out of the suspension.

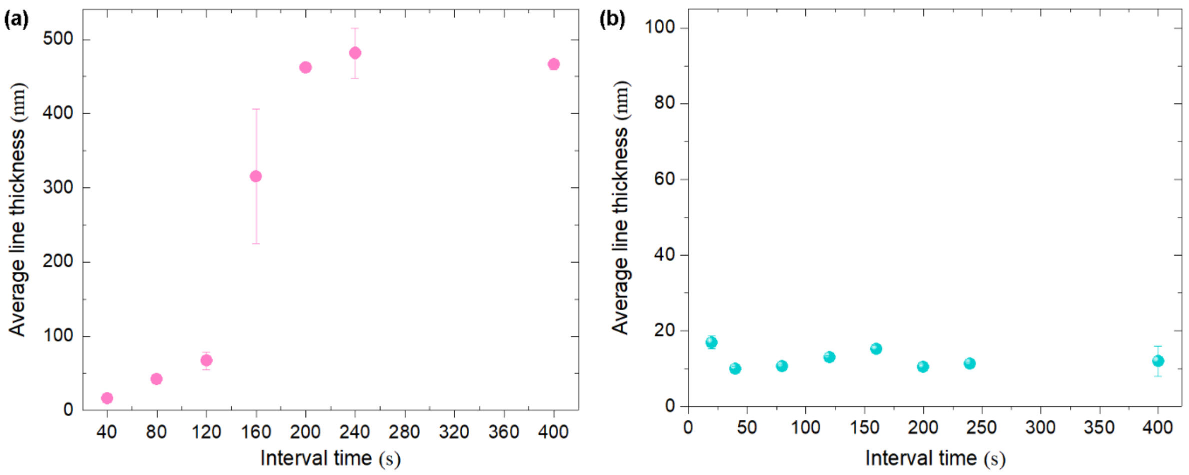

Figure S8. Average line thickness as a function of the interval time. (a) Line width 40 μm. (b) Line width 7.5 μm

Besides increasing the line thickness, the multiple time assembly process can help suppress the well-known "coffee-ring" effect[1] which is commonly encountered during drying of a liquid droplet. The solvent evaporation rate is non-uniform during the droplet drying process, higher at the edge of the droplet and lower at the center. The preferential evaporation at the edge induces an outward capillary flow, which carries nanoparticles towards the edge of the droplet, resulting in a ring-like accumulation of nanoparticles (**Figure S9**a). For the silver line assembled for only one time, a "coffee-ring" pattern with thick edges is formed, as exhibited in Figure S9c. However, when increasing the assembly times, the "coffee-ring" pattern disappears (Figure S9c). The reason for the disappeared "coffee-ring" pattern is two-fold. Firstly, the nanoparticles assembled in the previous steps increase the surface roughness in the pattern region, which suppresses the outward migration of nanoparticles in the following assembly steps (Figure S9b).[2] Secondly, the curved morphology of the assembled nanoparticles also suppresses the outward migration of nanoparticles.

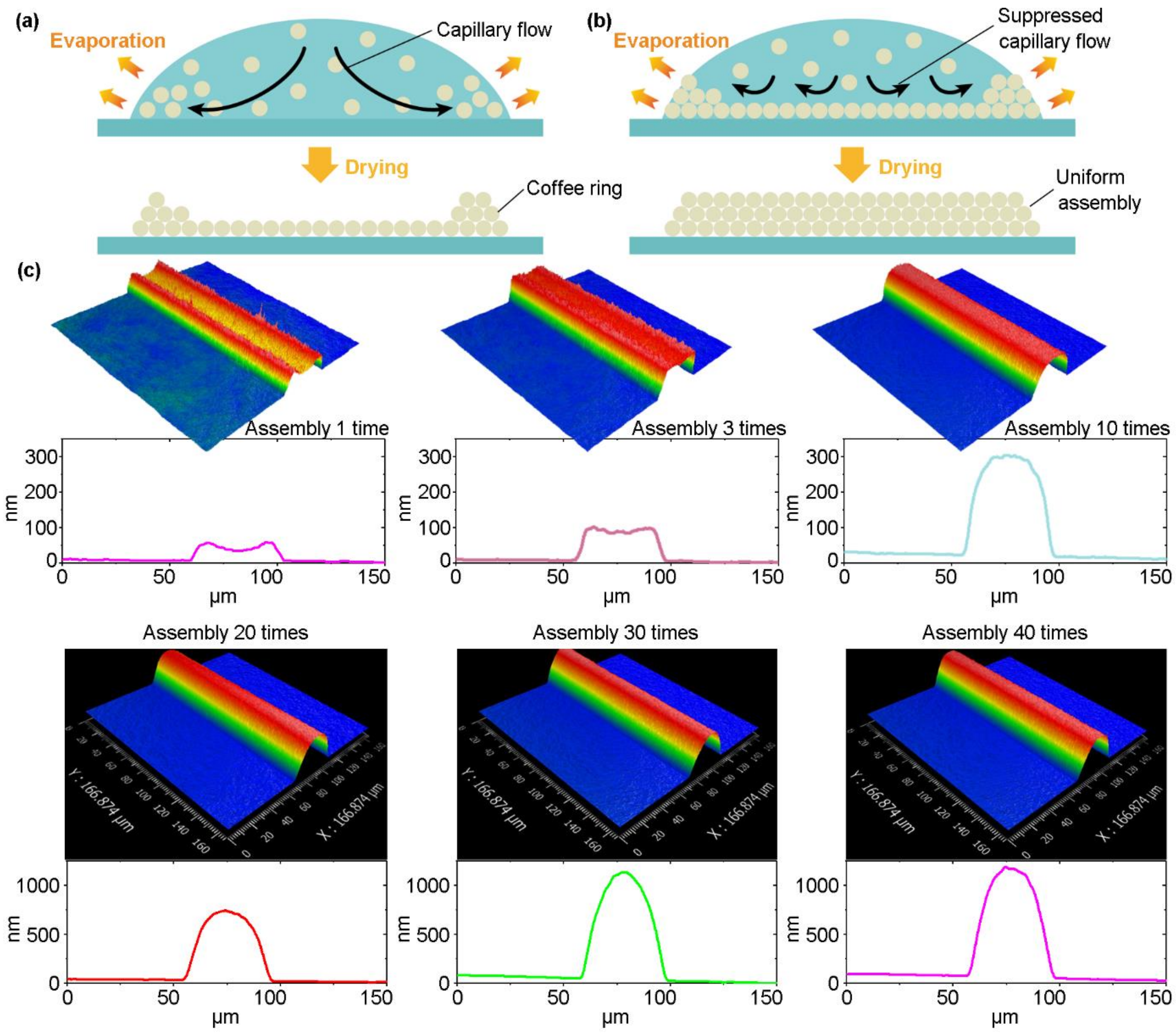

Figure S9. (a) Schematic illustration of the capillary flow during drying of a droplet on a flat surface and the resulting "coffee-ring" pattern. (b) Schematic illustration of the capillary flow during drying of a droplet on a rough and curved surface with assembled nanoparticles and the resulting pattern absent of the "coffee-ring". (c) 3D morphologies and cross-sectional profiles of silver lines with a width of 40 μm assembled for different times.

The resistivity of the assembled silver patterns was characterized by the four-probe method. To probe the silver patterns, four aluminum electrodes are fabricated using a combination of photolithography, thermal evaporation and lift-off processes. The layout of the four-probe test structure is exhibited in **Figure S10**a, wherein the blue patterns are assembled silver patterns and the green patterns are aluminum electrodes. Figure S10b shows a micrograph of one silver pattern as well as four aluminum electrodes. During the four-probe

measurement, a variable current is applied between electrodes 1 and 2, and a voltage is measured between electrodes 3 and 4. The measured voltage as a function of the applied current is plotted in Figure S10d. The slope of the voltage-current curve is the resistance of the silver pattern between electrodes 3 and 4. Using the Ohm's law, the resistivity of the silver pattern can be calculated as

$$\rho = \frac{tw}{l}\frac{V}{I} \qquad (1)$$

where t and w are the thickness and width of the silver pattern, l is the length of the silver pattern between electrodes 3 and 4, V and I are the voltage and current. The width, length and thickness of the silver patterns are 40 μm, 100 μm and 160 nm, respectively. The spacing between two adjacent aluminum electrodes is 20 μm. According to Equation 1, the resistivity of the silver pattern is calculated to be 1.12×10^{-5} Ω cm.

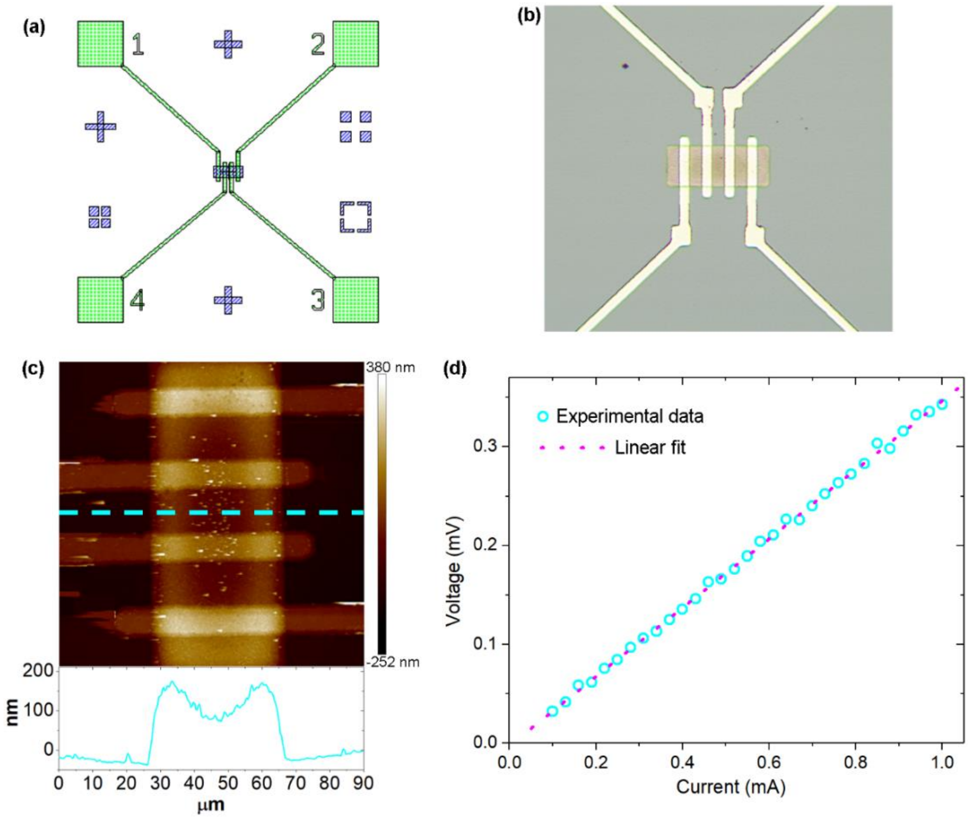

Figure S10. (a) Layout of the four-probe test structure. (b) Micrograph of one silver pattern as well as four aluminum electrodes. (c) AFM image of one silver pattern and four aluminum electrodes. The dashed line is a cross-sectional profile of the silver pattern, from which the

thickness of the silver pattern can be obtained. (d) Measured voltage between electrodes 3 and 4 as a function of the applied current.

Comparation of the efficiency of the two-step SEDA process and the conventional micro/nano fabrication process

Usually, it takes around half an hour to prepare functionalized substrates. For the two step SEDA process, the functionalized substrates need to be prepared twice. Therefore, the total time for the template preparation is around 1 hour. However, it should be mentioned that the functionalized substrates could be prepared in a much cheaper and rapid way. By using the microcontact printing process, the functionalized substrates can be prepared in tens of seconds.[3] The microcontact printing process is compatible with roll-to-roll process.[4] Therefore, high efficiency, low cost and large area fabrication of functionalized substrates should not be an issue.

During the SEDA process, a functionalized substrate is dipped into a nanoparticle suspension and then withdrawn at a speed of 6 mm/s. It should be mentioned that 6 mm/s is the maximum speed that could be applied by the dip coater in our lab. A higher withdraw speed is desired in order to further increase the assembly efficiency and would be investigated in the future. For a substrate 100 mm × 100 mm in size, it takes ~33 s (100/6*2) for one SEDA assembly step.

The drying time depends on the volume of entrained nanoparticle suspension during the SEDA process. According to the Darhuber model, a lower withdraw speed or a smaller line width result in a smaller volume of entrained suspension. At a line width of 40 μm and a withdraw speed of 6000 μm/s, a minimum drying time of 200 s is required. However, for narrower lines or smaller withdraw speeds, the minimum drying time will be much shorter. As we described above (Figure S8), for lines with width equal to or thinner than 7.5 μm, no time interval is needed as the entrained suspension dries immediately once the sample is pulled out of the suspension.

For silver lines 40 μm in width, the SEDA process should be conducted for 10 times to get a thickness of ~ 400 nm. The total time including the substrate preparation time (1800 s), assembly time (33*10=330 s) and drying time (200*10=2000 s) is 4130 s. To get a silver mesh with a line width of 40 μm, the SEDA process should be conducted twice. The total time will be 8260 s (2.3 h). However, if the line thickness decreased to 100 nm, the total time will be shortened to 4,765 s (1800+33*10/4+200*10/4=4,765 s=1.32 h). In this situation, the sheet resistance will be around 7 Ω/□, which is still smaller than that of ITO film (10 Ω/□).

For silver lines 7.5 μm in width, the SEDA process should be conducted for 40 times to get a thickness of ~ 400 nm. However, the interval drying time will be much shorter (20 s). The total time including the substrate preparation time (1800 s), assembly time (33*40=1320 s) and drying time (20*40=800 s) is 3,920 s. To get a silver mesh with a line width of 7.5 μm, the SEDA process should be conducted twice. The total time will be 7,840 s (2.2 h). Similarly, if the line thickness decreased to 100 nm, the total assembly time will be shortened to 4,660 s (1.29 h).

From the above calculation, it can be seen that most of the time is consumed to prepare the functionalized substrate and to dry the entrained nanoparticle suspension. If the microcontact printing process can be utilized, the substrate preparation time could be shortened significantly. If the drying time can be shortened by using volatile solvents, the total assembly time will also be much shorter. Another solution to decrease the assembly time is to increase the concentration of the nanoparticles in the suspension. In this way, more particles will be assembled in each time and thus the assembly time can be decreased.

As a comparation, the time to fabricate silver meshes using the conventional micro/nano fabrication process is calculated. For the conventional micro/nano fabrication process, the fabrication time is the same for 40 μm and 7.5μm line widths. The total time including the photolithography time (1800 s), film deposition time (7200 s.) and lift-off time (1800 s) is 10,800 s (~ 3 h). For the conventional micro/nano fabrication process, most of the time is

consumed in the film deposition process. The deposition time is mainly consumed on pumping down the chamber. For different film thickness, the total deposition time does not vary much. Table S1 compares the total fabrication time for silver meshes 100 nm in line thickness and 7.5 μm and 40 μm in line width. From the table, it can be seen that the two-step SEDA process is more efficient than the conventional micro/nano fabrication process.

Table S1. Total fabrication time for silver meshes 100 nm in line thickness and 7.5 μm and 40 μm in line width

	Two-step SEDA process	Conventional micro/nano fabrication process
Line width 40 um, line thickness 100 nm	4,765 s (1.32 h)	10,800 s (3 h)
Line width 7.5 um, line thickness 100 nm	4,660 s (1.29 h)	10,800 s (3 h)

Figure S11 shows detailed geometrical parameters of the silver lines for the capacitive-type touch screen panel (TSP). The silver lines are 10 μm in width and 200 μm in pitch. The length and number of the silver lines could be seen in Figure S11. The unit for all parameters is millimeter.

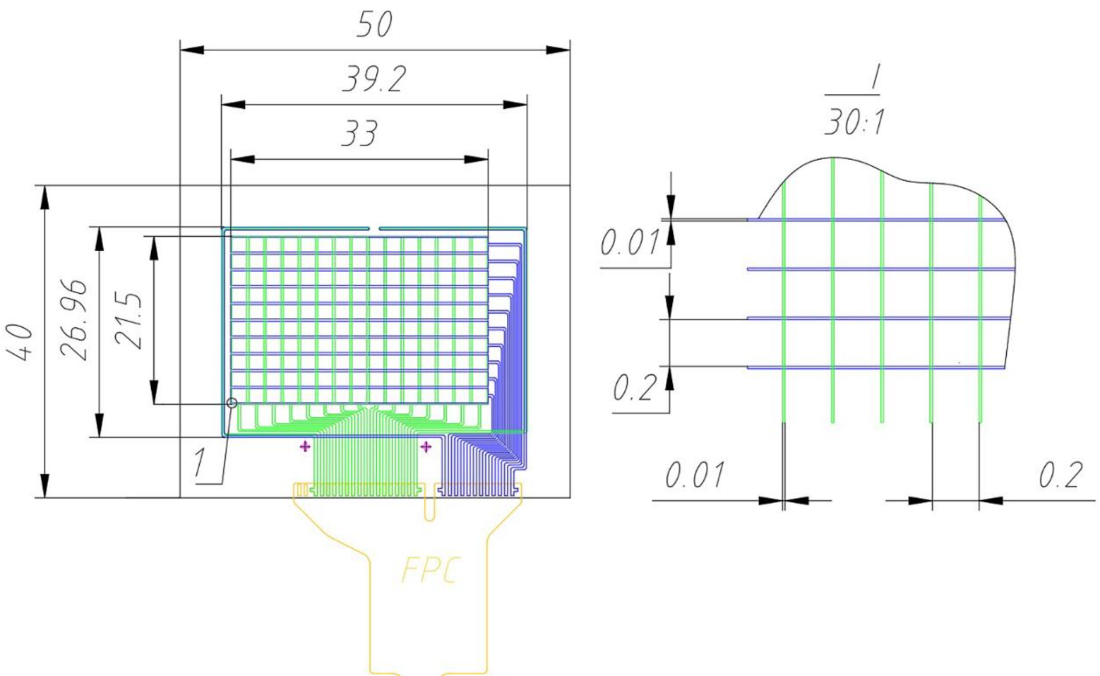

Figure S11. Detailed geometrical parameters of the silver lines for the capacitive-type TSP.

Figure S12 exhibits the stability of the fabricated TH during long-term thermal cycles at an applied voltage of 3 V. No significant change in heating performance was observed after 21 thermal cycles, demonstrating the excellent stability.

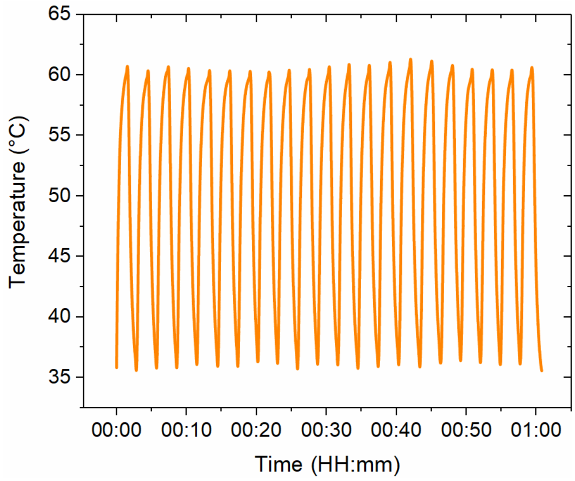

Figure S12. Long-term thermal cycles of the fabricated TH at an applied voltage of 3 V

Reference

[1] D. D. Robert, B. Olgica, F. D. Todd, H. Greb, R. N. Sidney, A. W. Thomas, *Nature* **1997**, 389, 827.

[2] L. Li, W. Li, Q. Sun, X. Liu, J. Jiu, M. Tenjimbayashi, M. Kanehara, T. Nakayama, T. Minari, *Small* **2021**, 17, 2101754.

[3] A. Kumar, H. A. Biebuyck, G. M. Whitesides, *Langmuir* **1994**, 10, 1498.

[4] J. A. Rogers, Z. Bao, A. Makhija, P. Braun, *Adv. Mater.* **1999**, 11, 741.
